# Ketalization of carbohydrate-derived levulinic esters using cellulose sulfuric acid as a heterogeneous catalyst: a closed-loop biorefinery approach†

**DOI:** 10.1039/d5ra00610d

**Published:** 2025-04-09

**Authors:** Poornachandra Shamanna Prabhakar, Saikat Dutta

**Affiliations:** a Department of Chemistry, National Institute of Technology Karnataka (NITK) Surathkal Mangalore-575025 India sdutta@nitk.edu.in

## Abstract

Levulinic ester ketals (LEKs) are carbohydrate-derived renewable chemicals with potential applications as chemical building blocks, fuel additives, solvents, monomers, and plasticizers. This work reports the synthesis of ethyl levulinate ethylene glycol ketal (LEK 1) and ethyl levulinate propylene glycol ketal (LEK 2) by the ketalization of carbohydrate-derived ethyl levulinate using cellulose sulfuric acid (CSA) as an efficient and recyclable heterogeneous acid catalyst. Cyclohexane was used as the solvent to remove water formed in the reaction by azeotropic distillation, and nearly quantitative isolated yields of LEK 1 and LEK 2 were obtained under optimized parameters. The transformation was optimized on various process parameters, and the CSA catalyst was successfully recycled. Using CSA as a catalyst for producing LEKs shows promise for a closed-loop carbohydrate-centric biorefinery approach.

## Abstract 2

Levulinic ester ketals (LEKs) are carbohydrate-derived renewable chemicals with potential applications as chemical building blocks, fuel additives, solvents, monomers, and plasticizers.

## Graphical abstract

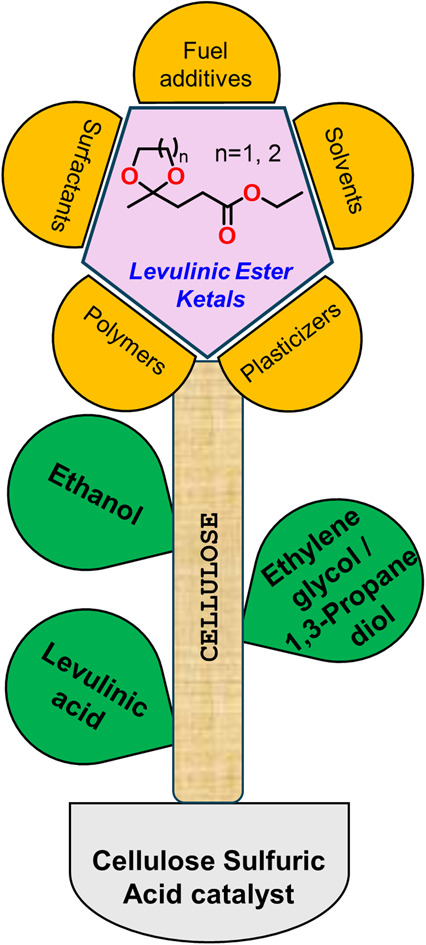

## Introduction

Cellulosic biomass has shown promise as a biogenic carbon-based renewable substitute for fossilized resources (*e.g.*, petroleum) for synthesizing liquid transportation fuels, organic chemicals, and synthetic polymers.^1^ In a carbohydrate-centric biorefinery, the carbohydrate content in biomass is converted into organic chemicals of desired molecular structure and properties, such as biofuels, fine chemicals, polymers, and other value-added products.^2^ The catalytic conversion of carbohydrates into organic chemicals has received particular attention since they are fast, selective, scalable, biomass-agnostic, and integrable in the preexisting petrorefinery infrastructure.^3,4^ Cellulose is the most abundant biopolymer, with an annual production of around 170 billion metric tons worldwide, and only a fraction of it is currently used for producing chemicals and materials.^5^ The non-food cellulosic biomass is often part of many waste streams (*e.g.*, agricultural residue, forestry waste, municipal wet waste).^6^ Cellulose and other carbohydrates can be catalytically transformed into organic chemicals following a two-step approach. In the first step, carbohydrates are converted to a handful of small organic compounds known as ‘platform chemicals’.^7^ The platform chemicals are then synthetically transformed into organic compounds of desired molecular structure and properties, preferably following catalytic routes. Levulinic acid (LA) is a well-documented carbohydrate-derived platform chemical that was listed as one of the top-twelve biomass-derived chemical building blocks by the National Renewable Energy Laboratory (NREL), USA,^8^ and also in the revised list published by Bozell and Petersen.^9^ LA is produced by the dehydration of hexose sugars (*e.g.*, glucose, fructose), polymeric carbohydrates of terrestrial origin (*e.g.*, inulin, starch, cellulose) and also marine carbohydrates (*e.g.*, carrageenan and chitin).^10^ LA can also be sourced from pentosans *via* the intermediacy of furfuryl alcohol.^11^ LA has been transformed into a wide array of organic chemicals with applications spanning from hydrocarbon fuels to fuel oxygenates to solvents to plasticizers to polymers to agrochemicals to pharmaceuticals.^12^ The literature on the production and derivative chemistry of LA has been periodically reviewed.^13–15^ The levulinic ester ketals (LEKs), produced by the ketalization of levulinic esters under acid catalysis, have numerous applications, including fuel additives, plasticizers, and surfactants.^16,17^ Wang *et al.* reported the synthesis of levulinic acid pentaerythritol ketal ester (LAPKE) of LA as a novel, biobased plasticizer for poly(vinyl chloride) (PVC).^18^ The two-step synthesis started with the Fischer esterification of LA with pentaerythritol. In the second step, the ester was subjected to ketalization by ethylene glycol (EG) using cyclohexane as the water removal agent. Sinisi *et al.* also synthesized a series of LEKs and examined their efficacy as plasticizers for PVC.^19^ LA was initially esterified by carboxylic acids of different alkyl chain lengths using *p*-toluenesulfonic acid (*p*TSA) as the acid catalyst. In the second step, the levulinic esters were ketalized by 1,3-propanediol (PDO). Gundekari *et al.* reported using Na-β-zeolite as a heterogeneous acid catalyst for the ketalization of ethyl levulinate (EL) with biorenewable glycerol.^20^ A 99% yield of the corresponding ethyl levulinate glyceral ketal was obtained using cyclohexane as the water removal agent. Segetis (now GF Biochemicals) has reported the synthesis of several LEKs in the patent literature.^21,22^

**Scheme 1:**
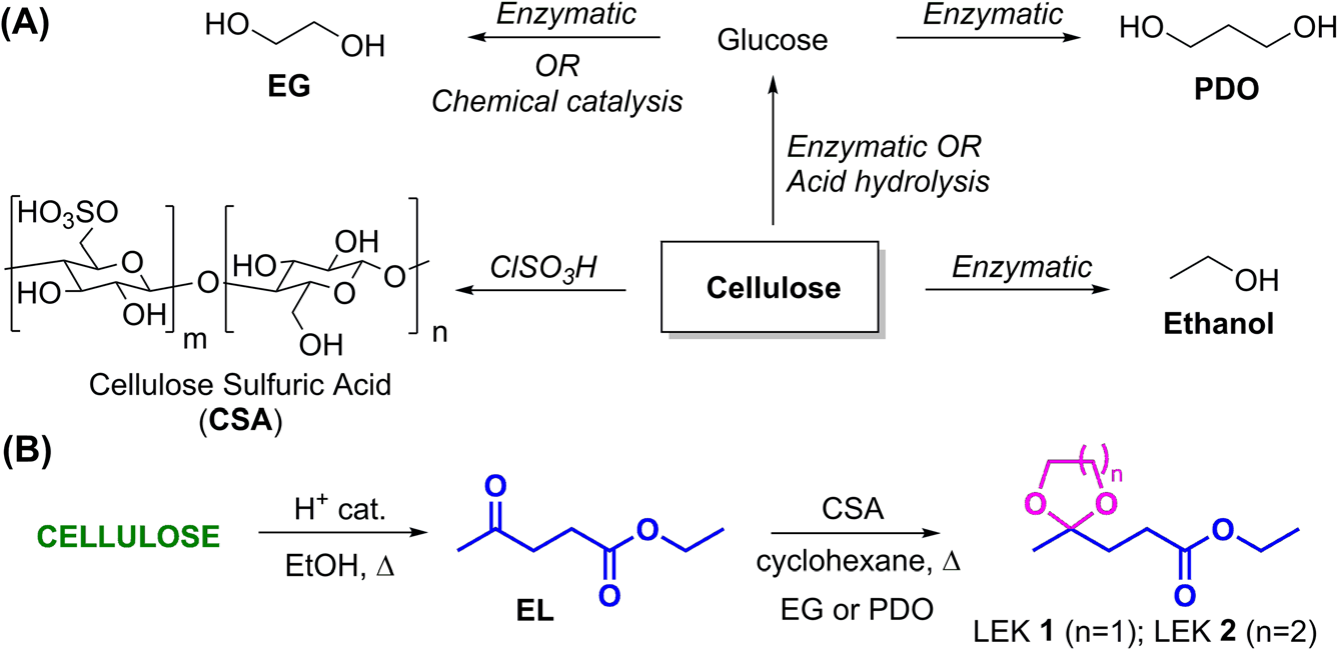
Sustainable synthesis of levulinic ester ketals from biomass-derived platform chemicals.

Interestingly, the reagents used in synthesizing LEKs are almost exclusively biorenewable in nature. LA and ethanol, required for synthesizing EL, are produced from the carbohydrate fraction of biomass. Ethanol is commercially produced by the enzymatic route, but the catalytic route has also received significant attention.^23^ LA is primarily produced by the acid hydrolysis of carbohydrates.^24^ The reagents for ketalization, such as EG and PDO, are also produced from carbohydrates following enzymatic or chemical-catalytic routes (Scheme 1A).^25^ Even though various homogeneous and heterogeneous acid catalysts have been explored for synthesizing LEKs, there is a constant search for inexpensive, active, selective, and eco-friendly acid catalysts that afford satisfactory yields of LEKs under economically feasible and environmentally acceptable conditions. Since ketalization of levulinic esters is a reversible reaction, the water byproduct must be removed from the reaction mixture to ensure excellent yields of the LEKs. Solvents like toluene and cyclohexane are frequently employed to remove water produced *in situ* by azeotropic distillation.^20^

Cellulose sulfuric acid (CSA), produced by anchoring sulfonic acid functionality with the free hydroxyl groups of cellulose, has shown promise as a non-toxic, non-hygroscopic, biodegradable, and recyclable heterogeneous acid catalyst for various organic transformations and multicomponent reactions.^26–28^ This work reports the synthesis of ethyl levulinate ethylene glycol ketal (LEK 1) and ethyl levulinate propylene glycol ketal (LEK 2) by the ketalization of EL with EG and PDO, respectively, using CSA as the acid catalyst (Scheme 1B). This study of synthesizing LEKs can be considered a closed-loop biorefinery approach since cellulose can be used as the sole feedstock for synthesizing the catalyst and all the starting materials (*i.e.*, LA, ethanol, EG) required for LEKs.^29^

## Experimental section

### Materials

Ethyl levulinate (>98%) was purchased from TCI Pvt. Ltd. Cyclohexane (99.5%) was purchased from Merck. Whatman filter paper (grade 5) was purchased from Cytiva. Microcrystalline cellulose was purchased from Sigma Aldrich. Chlorosulfonic acid (98%) was purchased from Spectrochem. Ethylene glycol (99%), chloroform (99%), ethyl acetate (99%), and sodium sulfate (anhydrous, 99%) were purchased from Loba Chemie Pvt. Ltd. Chloroform was dried over activated molecular sieves (4 Å) for 24 h before use. All the other chemicals were used without further purification.

### Characterization methods

The synthesized products were characterized by spectroscopic methods and matched with literature data. Fourier transform infrared (FTIR) spectra were collected using the ATR method in a Bruker Alpha II FTIR instrument equipped with zinc selenide (ZnSe) as the prism material. The compounds were dissolved in dichloromethane, and a thin film was made by evaporating a drop of the solution on the ATR counter. The FTIR spectra were averaged by collecting 24 scans at a scanning speed of 4 scans per second in the 500–4000 cm^−1^ range. For nuclear magnetic resonance (NMR) spectroscopy, the ^1^H-NMR spectra were collected in a Bruker NanoBay® NMR instrument at the operating radio frequency of 400 MHz, and the ^13^C-NMR spectra were recorded in the same instrument at the frequency of 100 MHz (calculated).

### Synthetic procedure

#### Preparation of CSA catalyst

CSA was prepared following a published literature.^30^ In a typical process, microcrystalline cellulose (5 g, 30.86 mmol) taken in a 100 mL round-bottomed flask was suspended in dry chloroform (20 mL). The flask was placed in an ice bath. A solution of chlorosulfonic acid (1.00 g, 8.58 mmol) in dry chloroform (10 mL) was added dropwise to the suspension of cellulose in chloroform under vigorous magnetic stirring. The stirring was continued for 2 h in an ice-water bath. The rapid evolution of HCl gas was observed during the reaction performed inside a fume hood, which allowed the gas to escape the flask. The reaction mixture was allowed to warm up to RT, and the stirring continued for an additional 2 h. The suspension was then filtered through a Whatman filter paper (grade 5), and the solid residue was washed with chloroform (5 × 10 mL). The resulting solid was then dried under vacuum for 6 h at RT. CSA was obtained as a white powder (5.24 g).

#### Synthesis of 3-(2-methyl-1,3-dioxolan-2-yl)propanoate (LEK 1)

Ethyl levulinate (1.00 g, 6.94 mmol) and ethylene glycol (0.540 g, 8.70 mmol) were taken in a round-bottomed flask (100 mL). Cyclohexane (15 mL) and the CSA catalyst (0.200 g, 20 wt% of EL) were added to the mixture. The flask was then connected to a Dean–Stark Apparatus. The flask was placed in a pre-heated (110 °C) oil bath and magnetically stirred continuously. The evolution of the reaction was monitored by measuring the volume of water collected in the graduated trap over time. The extent of conversion of EL was checked by thin-layered chromatography (TLC), and the reaction was stopped when EL was completely consumed. The completion of the reaction was confirmed by TLC analysis. The TLC plate was eluted with 2 : 1 (v/v) solvent mixture of petroleum ether and ethyl acetate, and was visualized using KMnO_4_ solution. The *R*_f_ value of EL and LEK 1 were calculated as 0.46 and 0.60, respectively. Upon completion of reaction, the reaction mixture was allowed to cool to RT. The reaction mixture was centrifuged, and the supernatant liquid was separated. The organic layer was gently washed with water in a separating funnel to remove excess ethylene glycol. The organic layer was dried over anhydrous Na_2_SO_4_ and then evaporated under reduced pressure in a rotary evaporator to get pure ethyl 3-(2-methyl-1,3-dioxolan-2-yl)propanoate (LEK 1) (1.278 g, 98%) as a light-yellow oil (*R*_f_ value = 0.59 in 2 : 1 (v/v) solvent mixture of petroleum ether and ethyl acetate). Characterization data: ^1^H-NMR (CDCl_3_, 400 MHz) *δ* (ppm): 4.08 (q, 2H, *J* = 7.2 Hz), 3.89 (q, 4H, *J* = 4.0 Hz), 2.33 (t, 2H, *J* = 7.2 Hz), 1.98 (t, 2H, *J* = 7.2 Hz), 1.27 (s, 3H), 1.20 (t, 3H, *J* = 7.2 Hz); ^13^C-NMR (CDCl_3_, 100 MHz) *δ* (ppm): 173.7, 109.3, 64.9, 60.4, 34.1, 29.2, 24.1, 14.3; FTIR (cm^−1^): 2982, 1733, 1135, 1048.

#### Synthesis of ethyl 3-(2-methyl-1,3-dioxan-2-yl)propanoate (LEK 2)

The same synthetic protocol was followed as described in the case of LEK 1, except propane-1,3-diol (0.662 g, 8.70 mmol) was used instead of ethylene glycol. LEK 2 was obtained as a light yellow oil (1.317 g, 94%). Characterization data: ^1^H-NMR (400 MHz, CDCl_3_, *δ* ppm): 4.02 (q, 2H, *J* = 7.2 Hz), 3.84–3.71 (m, 4H), 2.32 (t, 2H, *J* = 8.0 Hz), 1.91 (t, 2H, *J =* 8.0 Hz), 1.69–1.63 (m, 1H), 1.46–1.41 (m, 1H), 1.28 (s, 3H), 1.14 (t, 3H, *J* = 7.2 Hz); ^13^C-NMR (100 MHz, CDCl_3_, *δ* ppm): 173.79, 98.20, 60.15, 59.71, 33.81, 28.54, 25.42, 20.55, 14.20; FTIR (cm^−1^): 2978, 1732, 1310, 1247.

### Recycling of the CSA catalyst

After the reaction completion, the reaction mixture of LEK 1 (or LEK 2) was cooled to room temperature. The CSA catalyst was recovered by filtration of the reaction mixture using Whatman filter paper (grade 5). The recovered catalyst was washed with ethyl acetate (10 mL × 3) and dried at 60 °C for 12 h in a hot-air oven before using in the next cycle. Alternatively, the reaction mixture was centrifuged, the CSA catalyst was washed with ethyl acetate (10 mL × 3), and subjected to the next catalytic cycle without the filtration and drying step.

### Estimation of acid sites in the CSA catalyst

CSA (0.1 g) was added into 10 mL of aqueous solution of KCl (0.1 M) and stirred for 30 min. The resulting solution was titrated by a standard solution of KOH (0.02 M), and the pH evolution was determined by a pH meter (EQUIPTRONICS Digital pH Meter Model EQ 610). The equivalence point of the analyte was determined. The number of acidic protons was calculated using the equivalence point (mL) against 0.02 M KOH. The calculated acidic protons present in 0.1 g of the catalyst was extrapolated to calculate the corresponding value for 1 g of the catalyst.

## Results and discussion

**Fig. 1:**
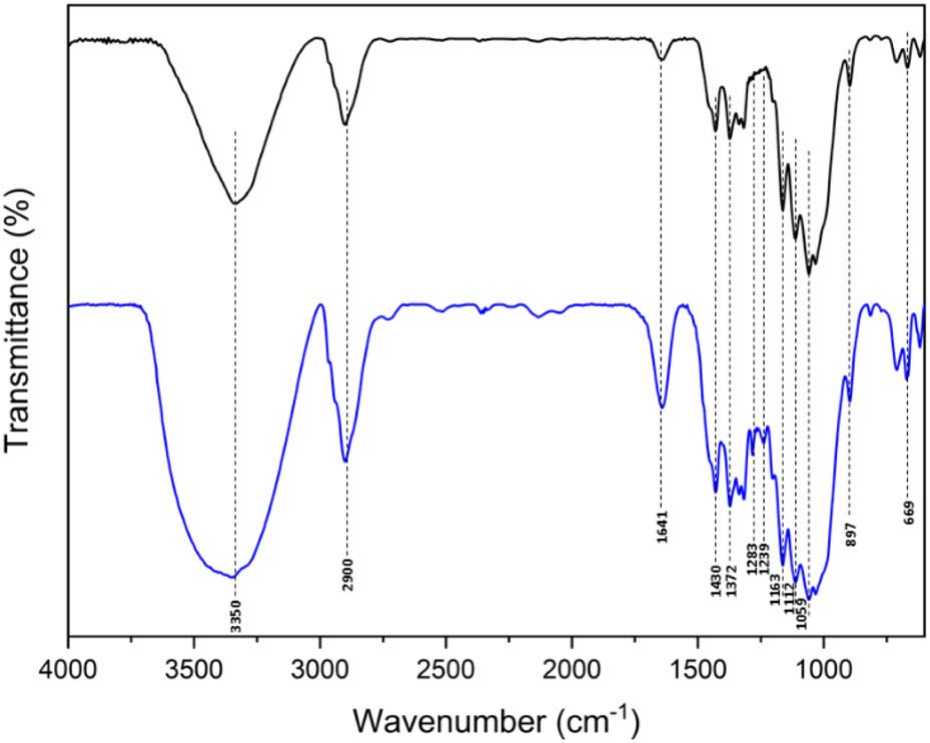
The overlay of FTIR spectra of microcrystalline cellulose (black) and CSA (blue).

The CSA catalyst prepared by using chlorosulfonic acid as the sulfonating agent was characterized by FTIR, PXRD, and elemental analysis data. The FTIR spectra (overlay) of microcrystalline cellulose and CSA are displayed in Fig. 1. The broad absorbance at 3350 cm^−1^ can be assigned to the O–H stretching vibration of the free hydroxyl groups in CSA. The peak is also contributed by the moisture strongly bound on the surface of the catalyst. The FTIR absorbance at 2900 cm^−1^ is due to C(sp^3^)–H stretching vibration peak of the glucose units in the cellulose chain. The absorbance at 1059 cm^−1^ is assigned to the C–O stretching vibration.^31^ The bands at 1283 cm^−1^ and 1239 cm^−1^ are attributed to the O=S=O asymmetric and symmetric stretching vibration. The absorption peak at 669 cm^−1^ is due to the bending frequency of the sulfonic acid group.^32^ The –OH vibration of sulfonic groups overlapped with the –OH vibration of the free hydroxyl groups in cellulose.

**Fig. 2:**
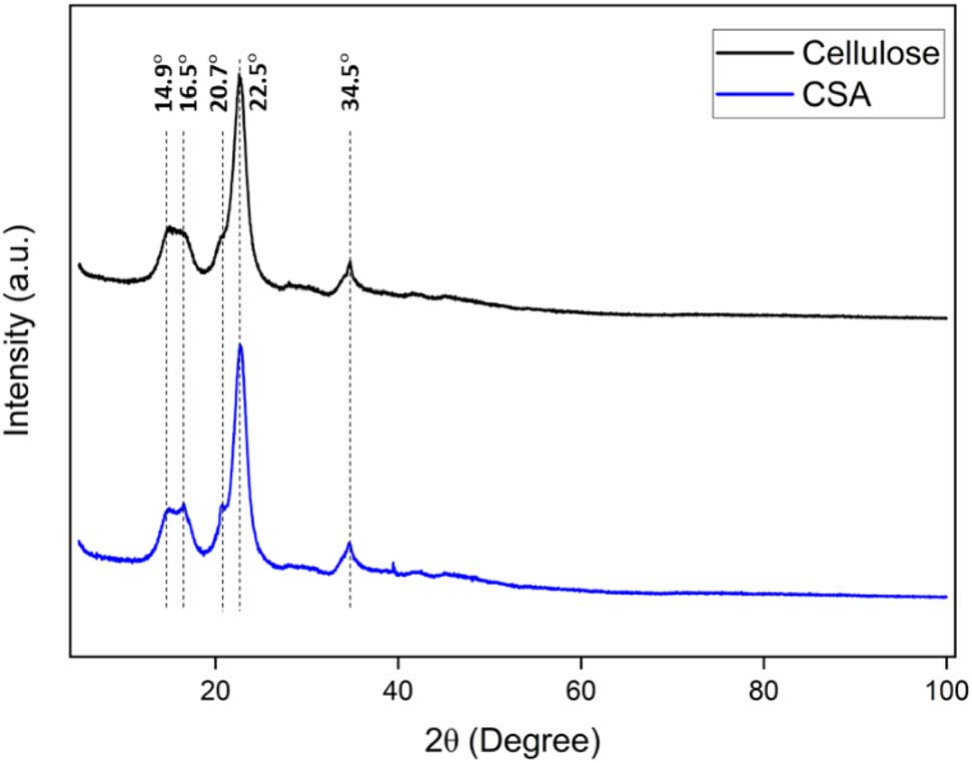
The overlay of PXRD of microcrystalline cellulose (black) and CSA (blue).

The powder X-ray diffraction pattern of the matrix showed characteristic diffraction peaks for the cellulose at 14.9°, 22.5° and 34.5°.^33^ The CSA catalyst has two peaks at 16.5° and 20.7°, respectively, in addition to the cellulose peaks. It shows that the original polymeric structure of cellulose is retained (Fig. 2). The sulfur content of the samples determined by conventional elemental analysis was 0.55 mmol g^−1^ for CSA. The concentration of acidic sites in CSA was determined to be 0.55 mmol g^−1^ by acid–base titration, which was the same as sulfur content. These results indicated that most of the sulfur content in the CSA sample was in the form of the sulfonic acid group.

**Fig. 3:**
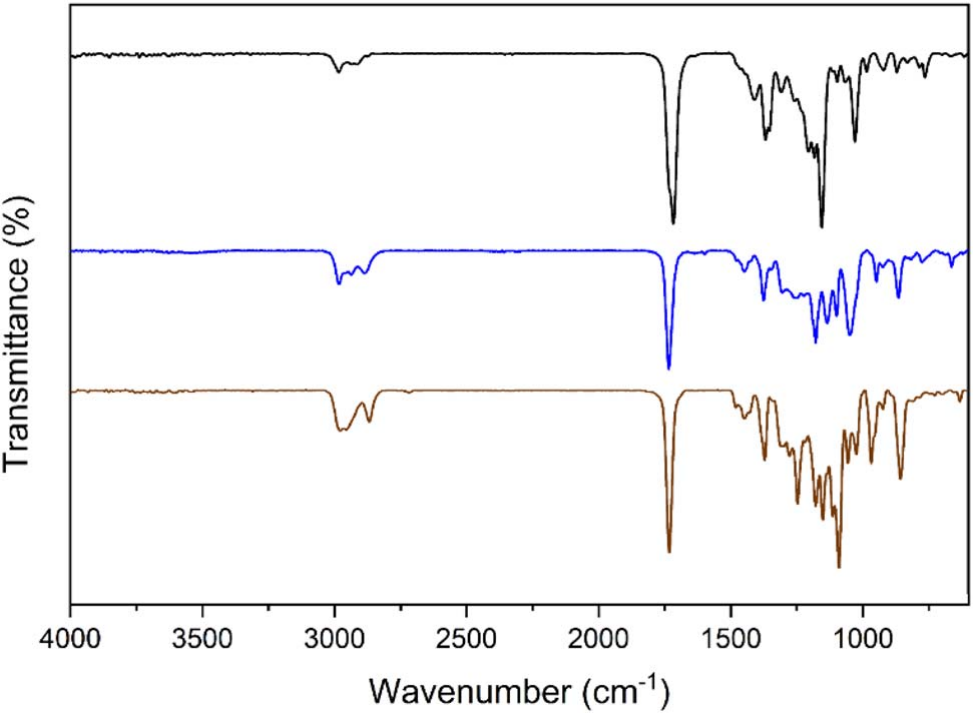
The overlay of the FTIR spectra of EL (black), LEK 1 (blue), and LEK 2 (brown).

Once the formation of CSA was confirmed, it was used for ketalization of EL using EG as the reagent. In our initial experiment, the ketalization of EL was carried out by condensing EL with an equimolar amount of EG in a round-bottomed flask equipped with a condenser at 100 °C. However, even after 24 h, the reaction showed only trace conversion. Increasing the catalyst loading to 20 wt% and the EG to 1.5 equivalents relative to EL also had no significant effect. The slow reaction kinetics and formation of multiple products in the reaction mixture is likely due to the presence of water, a benign byproduct, which could not only reverse the ketalization reaction but also hydrolyze the ester into acid or transesterification; thus, it was crucial to minimize the role of water in the system. To address this, a Dean–Stark apparatus was attached to the round-bottom flask to ensure its continuous removal during the reaction by azeotropic distillation in cyclohexane as a safe organic solvent. The reaction mixture with 10 wt% catalyst was then magnetically stirred in a pre-heated oil bath at 110 °C for 6 h. Upon completion, the mixture was filtered through Whatman filter paper (grade 5), and the solvent was evaporated to yield ethyl 3-(2-methyl-1,3-dioxolan-2-yl)propanoate as a light yellow oil. The identity and purity of the LEKs were confirmed by spectroscopic techniques (FTIR, ^1^H-NMR, and ^13^C-NMR). An overlay of the FTIR spectrum of EL, LEK 1, and LEK 2 is shown in Fig. 3.

**Fig. 4:**
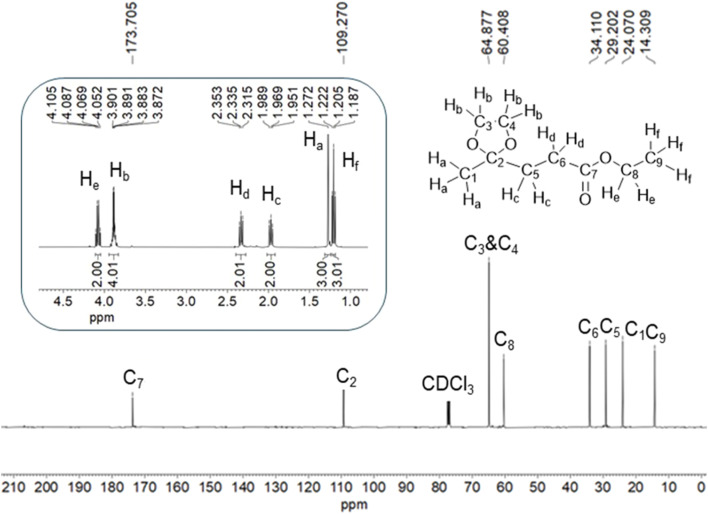
The ^1^H-NMR (inset) and ^13^C-NMR spectra of LEK 1 demonstrate the identity and purity of the synthesized compound.

The ^1^H-NMR and ^13^C{^1^H}-NMR spectra of LEK 1 are shown in Fig. 4. As expected, the ketonic carbon of EL appearing at 202.4 ppm disappeared in LEK 1.^34^ The ketalized carbon (C2) appears at 109.3 ppm. The methylene protons in EG in LEK 1 appear at 3.89 ppm in the ^1^H-NMR spectrum, whereas the corresponding carbon atoms (C_3_ & C_4_) appear at 64.9 ppm. The methyl carbon (C_1_) adjacent to the ketalized carbon is slightly upfield shifted. No significant change in the chemical shift of other protons or carbon atoms was observed in their corresponding spectrum. LEK 1 was also characterized by DEPT-135, and 2D-NMR techniques (^1^H–^1^H COSY & HMQC) (Fig. S4–S6, ESI†).

**Fig. 5:**
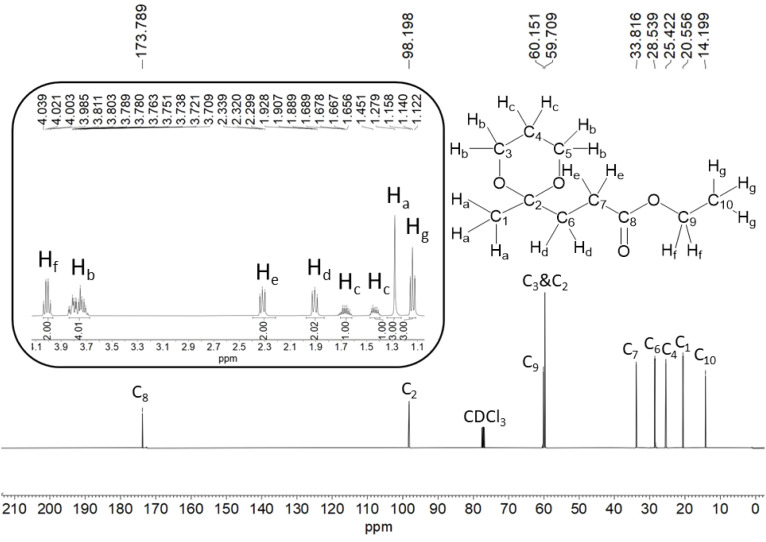
The ^1^H-NMR (inset) and ^13^C-NMR spectra of LEK 2 demonstrate the identity and purity of the synthesized compound.

The ^1^H-NMR and ^13^C{^1^H}-NMR spectra of LEK 2 are shown in Fig. 5. The ketalized carbon (C_2_) appears at 98.2 ppm. The methylene protons (H_b_) of PDO in the ^1^H-NMR spectrum LEK 2 appear as multiplet around 3.81–3.84 ppm. The methylene protons (H_c_) of PDO appeared as a multiplet at 1.41 ppm and 1.68 ppm. The C_3_ and C_5_ carbon appeared at 59.7 ppm, whereas the C_4_ carbon appeared at 25.4 ppm. The methyl carbon (C_1_) adjacent to the ketalized carbon appeared at 25.5 ppm, whereas the methyl carbon in the ethyl ester (C_10_) appeared most upfield at 14.2 ppm. LEK 2 was also characterized by DEPT-135, and 2D-NMR techniques (^1^H–^1^H COSY & HMQC) (Fig. S10–S12, ESI†).

Molar ratio of the two substrates (*i.e.*, EL and EG) was found to play crucial role in determining the extent of reaction (*i.e.*, conversion) and isolated yield of LEK 1. Initially, the reaction was carried out using an equimolar ratio of EL and EG in the presence of 10 wt% CSA catalyst. Even after 24 h of reaction duration, complete conversion of EL was not achieved. Increasing the EG amount to 1.25 equivalents of EL significantly improved the reaction kinetics, leading to complete conversion of EL within 6 h and a 98% yield of LEK 1. Increasing the equivalent of EG further to 1.5 equivalents of EL did not show any appreciable change in the reaction kinetics.

**Fig. 6:**
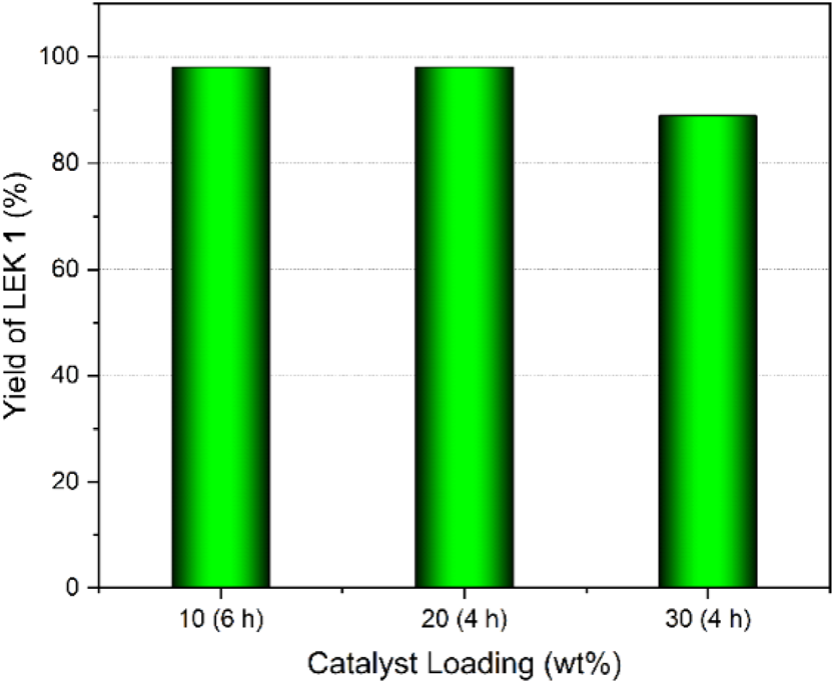
Effect of catalyst loading on the yield of LEK 1.

The catalyst loading plays a crucial role in reaction kinetics and product selectivity. The reaction was completed in 6 h using a 10 wt% (with respect to EL) CSA catalyst, yielding 98% of LEK 1 (Fig. 6). Raising the catalyst loading to 20 wt% further enhanced the reaction rate, completing in 4 h, while the yield of LEK 1 remaining virtually unaltered. Further increasing in the catalyst loading to 30 wt% showed a decrease in the product yield to 89%. A higher concentration of the acid catalyst in the reaction mixture promotes high conversion with lower selectivity, leading to the competition of ketalization with transesterification with the alcohol, forming some side products, such as the transesterification of EL with EG and the hydrolysis of EL into LA.^35^ When the loading of CSA was reduced to 5 wt%, the reaction took 15 h for complete conversion and afforded a 94% yield of LEK 1. Therefore, 20 wt% catalyst loading was considered the optimal amount for this reaction.

Under the optimized condition, other catalysts, such as *p*TSA, phosphotungstic acid (PTA), and Amberlyst-15(H), were also compared (Table 1). CSA catalyst showed comparable catalytic activities with other frequently used acid catalysts for the acetalization/ketalization reactions. Additionally, CSA, being a heterogeneous catalyst, could be recovered by filtration, washing, and drying. The control reaction (without catalyst) afforded no appreciable amount of product after 4 h at 110 °C (entry 5, Table 1) and even when the duration was extended to 10 h.

**Table 1:** Influence of acid catalysts on the synthesis of LEK 1 and LEK 2^a^

Entry	Catalyst	Yield of LEK 1	Yield of LEK 2
1	*p*TSA	98%	96%
2	PTA	85%	84%
3	Amberlyst-15(H)	88%	90%
4	CSA	98%	94%
5	No catalyst (control)	Trace	Trace

a Reaction conditions: EL (1.000 g), EG (0.540 g) or PDO (0.662 g), cyclohexane (20 mL), catalyst (20 wt%), 110 °C, 4 h.

**Fig. 7:**
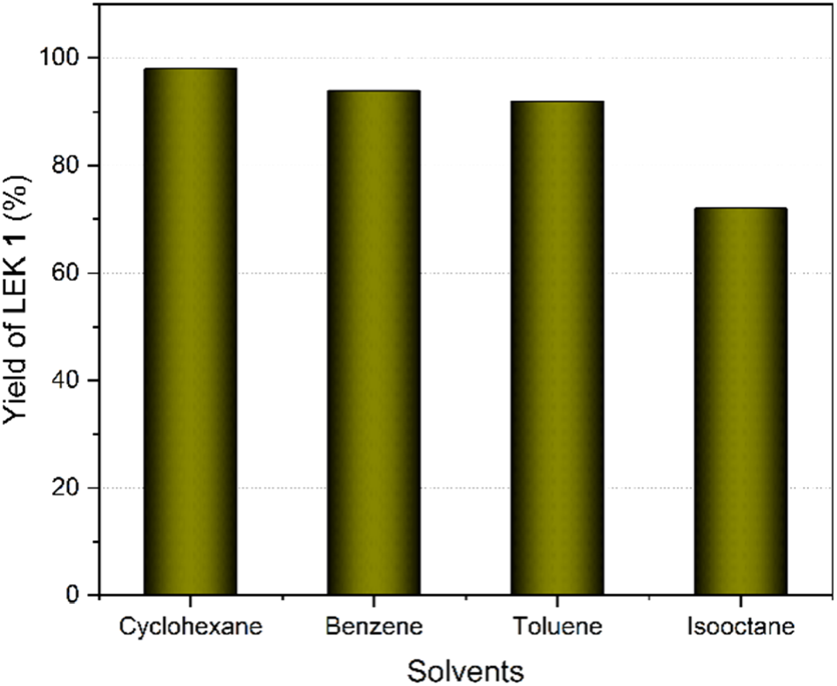
Solvent screening for synthesizing LEK 1. Reaction conditions: EL (1.000 g), EG (0.540 g), solvent (20 mL), CSA catalyst (20 wt%), 110 °C, 4 h.

Keeping all the optimized parameters constant, the effect of solvents was investigated (Fig. 7). When the reaction was carried out in cyclohexane, benzene, toluene, and isooctane as solvents, the yields of LEK 1 obtained were 98%, 94%, 92%, and 72%, respectively. Benzene, cyclohexane, and toluene provided comparable (>90%) yields, whereas a lower yield was observed with isooctane, likely due to reduced selectivity, leading to the formation of multiple side products *via* transesterification and hydrolysis of EL. Among the solvents studied, cyclohexane exhibited the highest efficiency and was selected as the optimal reaction medium for further studies.

**Fig. 8:**
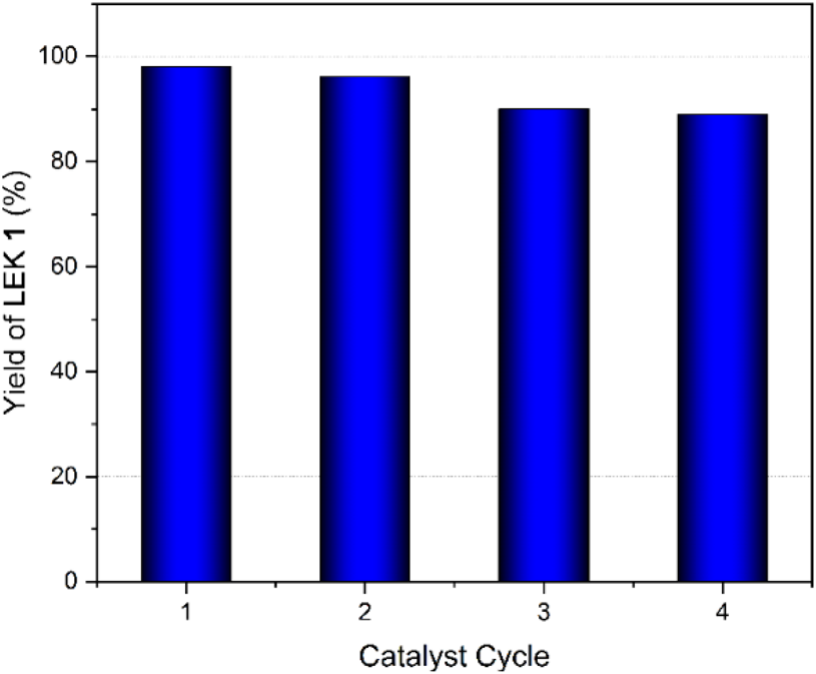
Recyclability test for the CSA catalyst during the synthesis of LEK 1.

The recyclability of the CSA catalyst was studied for the synthesis of LEK 1 (Fig. 8). The solid CSA catalyst was recovered from the crude reaction mixture by filtration through a filter paper or by centrifugation. The recovered catalyst was washed with ethyl acetate (10 mL × 3) and dried at 60 °C for 12 h in a hot-air oven. The drying step was optional since the catalytic activity did not alter noticeably by the drying step. These processes were repeated four times, and the reusability results of CSA showed that the catalyst was stable and retained its catalytic activity for four consecutive cycle. Subsequently, there was a gradual decrease in the product yield, which could be attributed to the leaching of the sulfonic acid group in the CSA catalyst. These results clearly indicated that the prepared CSA could be reused for four cycles without catastrophic loss in its catalytic activity. In fact, even after four cycles, the yield of LEK 1 was still excellent (∼90%). The catalyst may be reactivated by reintroducing the sulfonic acid functionality by reacting with chlorosulfonic acid.

## Conclusion

In conclusion, cellulose sulfuric acid (CSA) was successfully employed as an efficient and sustainable heterogeneous acid catalyst for the ketalization of ethyl levulinate with ethylene glycol and 1,3-propanediol. This process yielded the corresponding levulinic ester ketals (LEKs) in near quantitative yields under optimized conditions. Cyclohexane proved to be an efficient solvent for the azeotropic removal of the water byproduct. The catalytic activity and selectivity of the CSA catalyst were compared with commonly used homogeneous and heterogeneous acid catalysts. The CSA catalyst was successfully recycled and reused for four cycles, avoiding extensive purification and without catastrophic loss of catalytic activity. The optimized reaction conditions were applied to produce 10 g of LEK 1 to demonstrate the scalability of the process. Future studies will explore using levulinic acid as the feedstock for the step-economic and pot-economic preparation of LEKs.

## Data availability

The data supporting this article have been included as part of the ESI.† The ESI file contains spectra (FTIR, ^1^H-NMR, and ^13^C-NMR, DEPT-135, ^1^H–^1^H COSY, and HMQC), photographic image of the reaction setup, and image of the CSA catalyst (fresh & recycled).

## Author contributions

Poornachandra Shamanna Prabhakar performed the experiments, characterized the CSA catalyst and LEKs, and edited the manuscript. Saikat Dutta conceptualized the idea, supervised the work, and wrote the original manuscript.

## Conflicts of interest

The authors declare no competing interest.

## Supplementary Material

RA-015-D5RA00610D-s001
