# Supplementary material for: Ketalization of carbohydrate-derived levulinic esters using cellulose sulfuric acid as a heterogeneous catalyst: a closed-loop biorefinery approach
Source: RSC Adv. 2025 Apr 9;15(15):11301–7. doi: 10.1039/d5ra00610d (PMC11980415; doi:10.1039/d5ra00610d)
Supplement: RA-015-D5RA00610D-s001 [file RA-015-D5RA00610D-s001.pdf]

Supplementary Information (SI) for RSC Advances.
This journal is © The Royal Society of Chemistry 2025

Supplementary Information

Ketalization of carbohydrate-derived levulinic esters using cellulose sulfuric acid as a heterogeneous catalyst: A closed-loop biorefinery approach

Poornachandra S P [a] *and Saikat Dutta* [a*]

[a] Department of Chemistry, National Institute of Technology Karnataka (NITK), Surathkal, Mangalore-575025, Karnataka, India.

[*] Corresponding author. E-mail: sdutta@nitk.edu.in

Number of Pages: 09

Number of Figures: 15

Spectra of data of Ethyl 3-(2-methyl-1,3-dioxolan-2-yl)propanoate (LEK 1)[1]

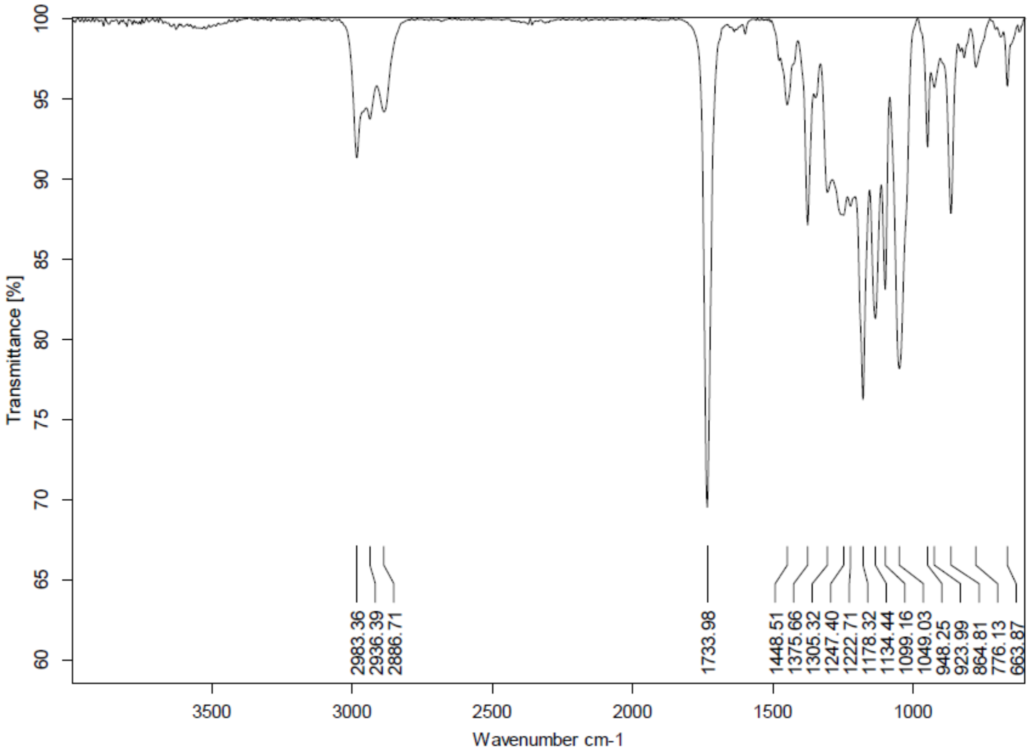

Figure S1. The FTIR spectrum of **LEK 1**.

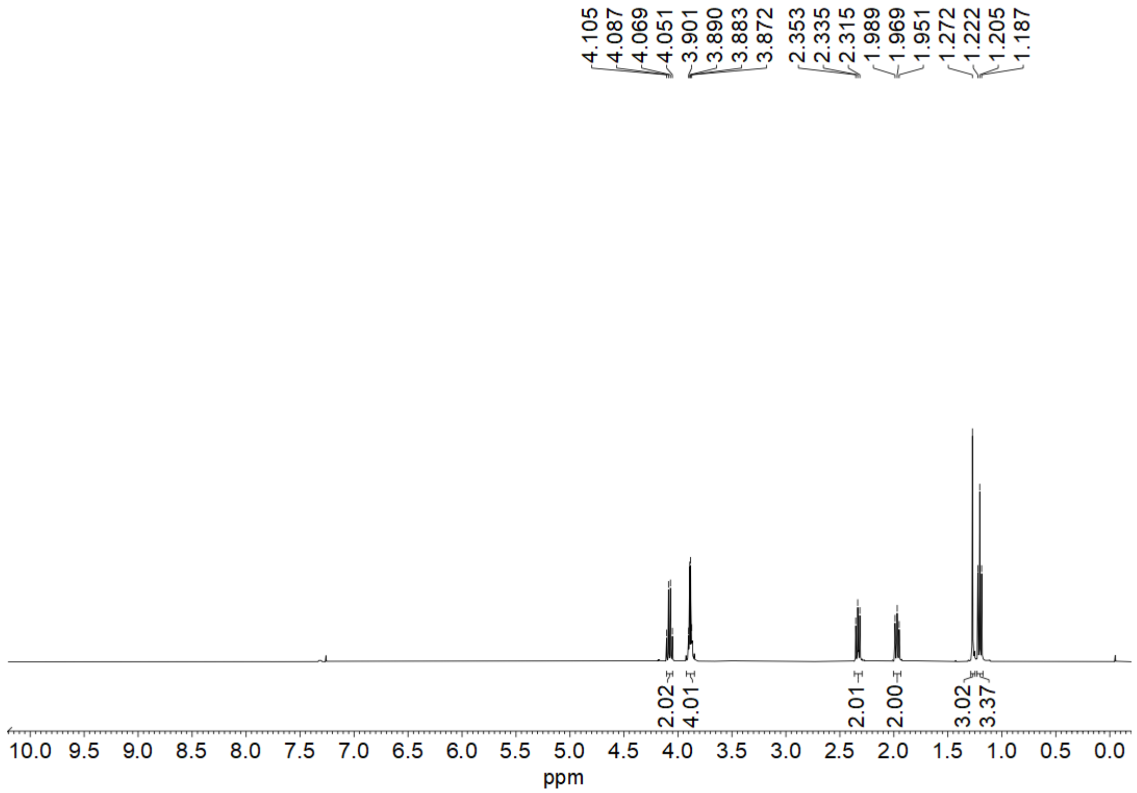

Figure S2. The ^{1}H-NMR spectrum of **LEK 1**.

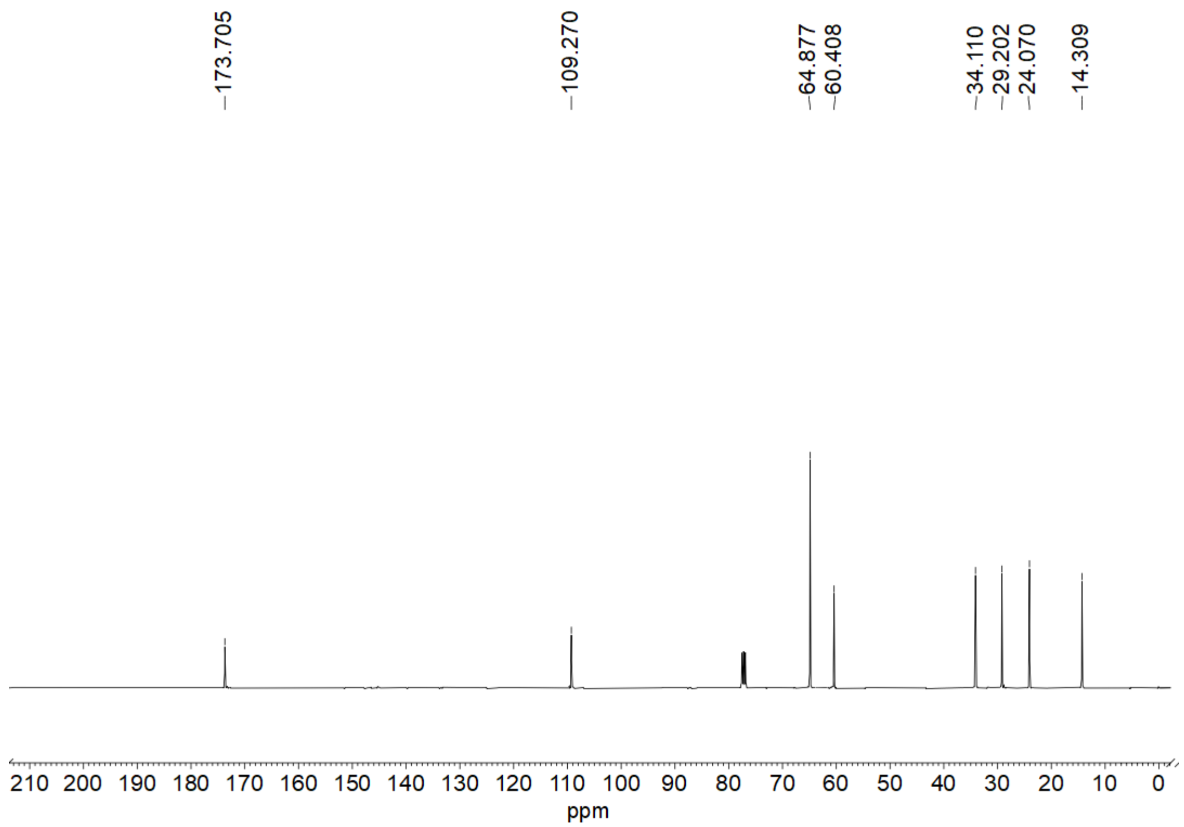

Figure S3. The ^{13}C-NMR spectrum of **LEK 1**.

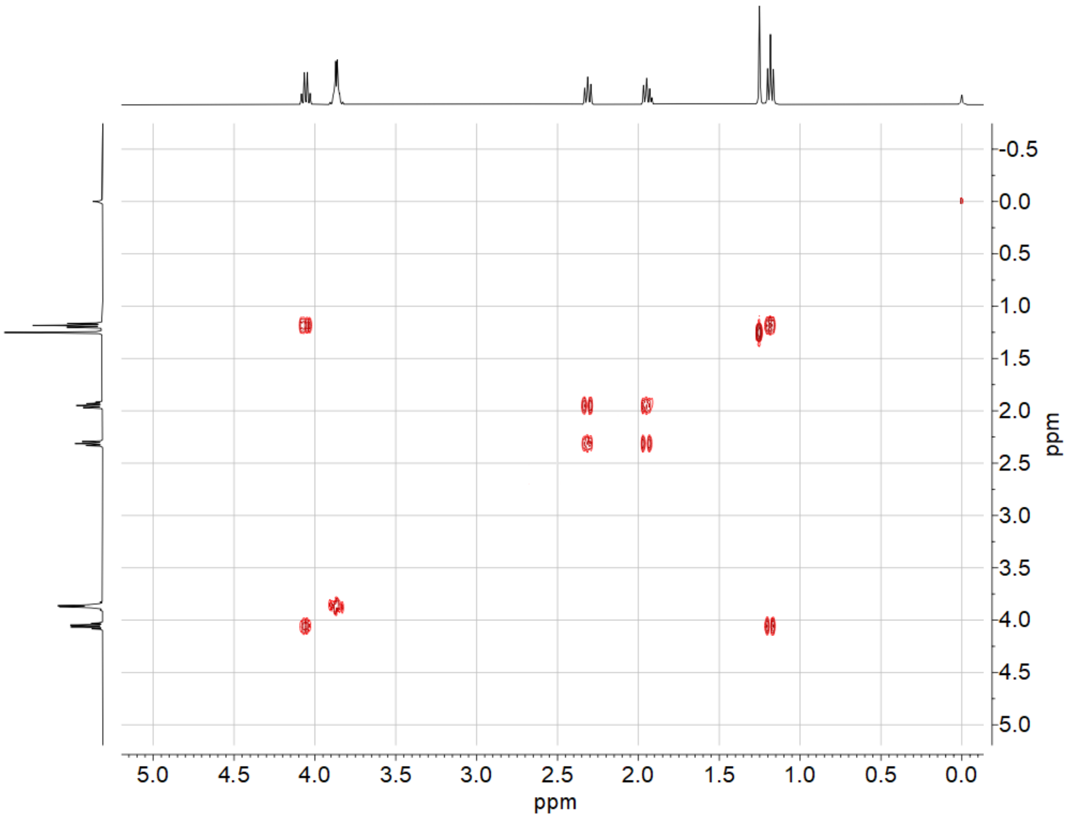

Figure S4. The ^{1}H-^{1}H COSY spectrum of **LEK 1**.

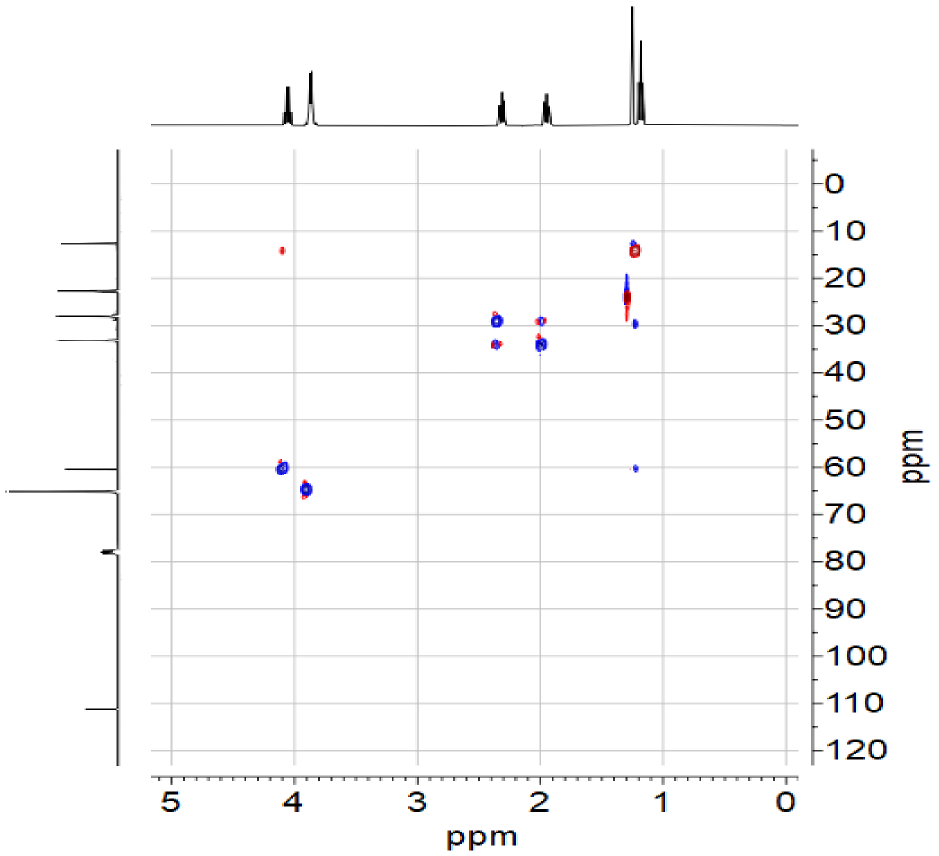

Figure S5. The HMQC spectrum of **LEK 1.**

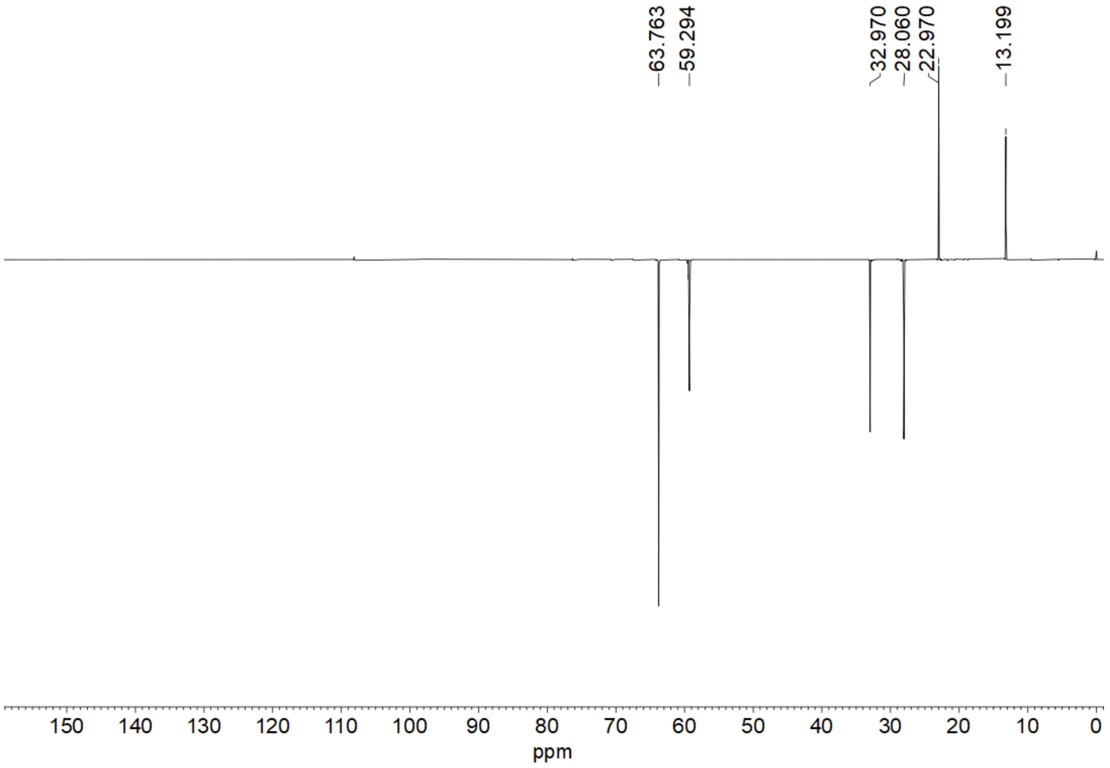

Figure S6. The DEPT-135 spectrum of **LEK 1**.

Spectra of Ethyl 3-(2-methyl-1,3-dioxan-2-yl)propanoate (LEK 2)[1]

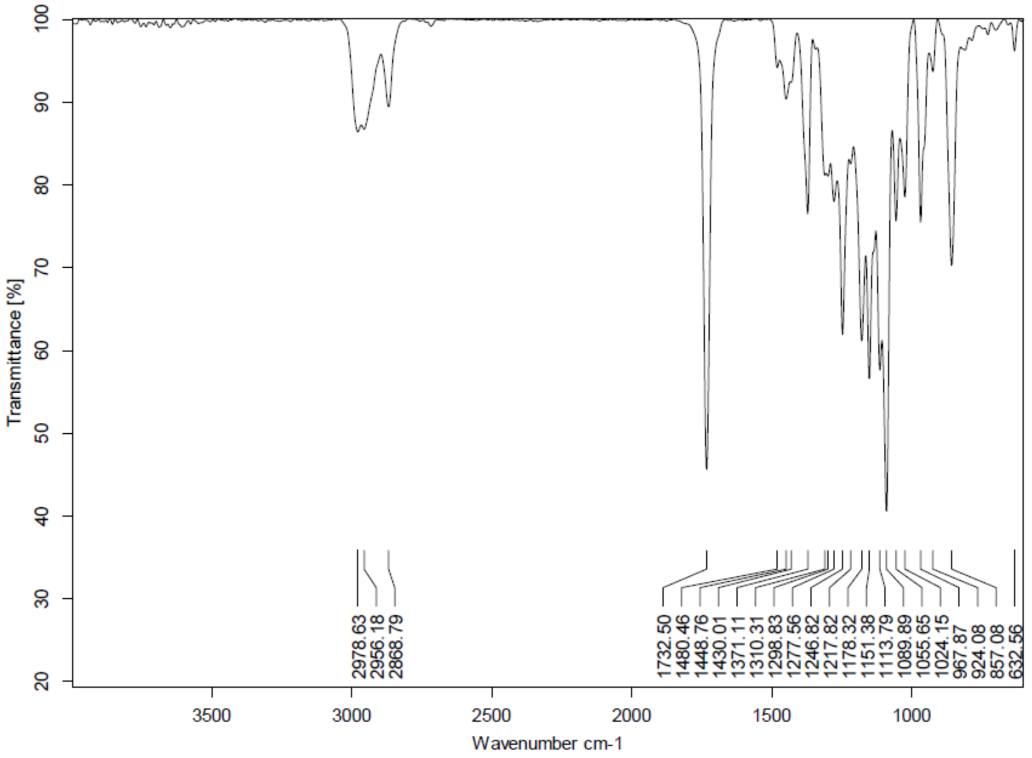

Figure S7. The FTIR spectrum of **LEK 2.**

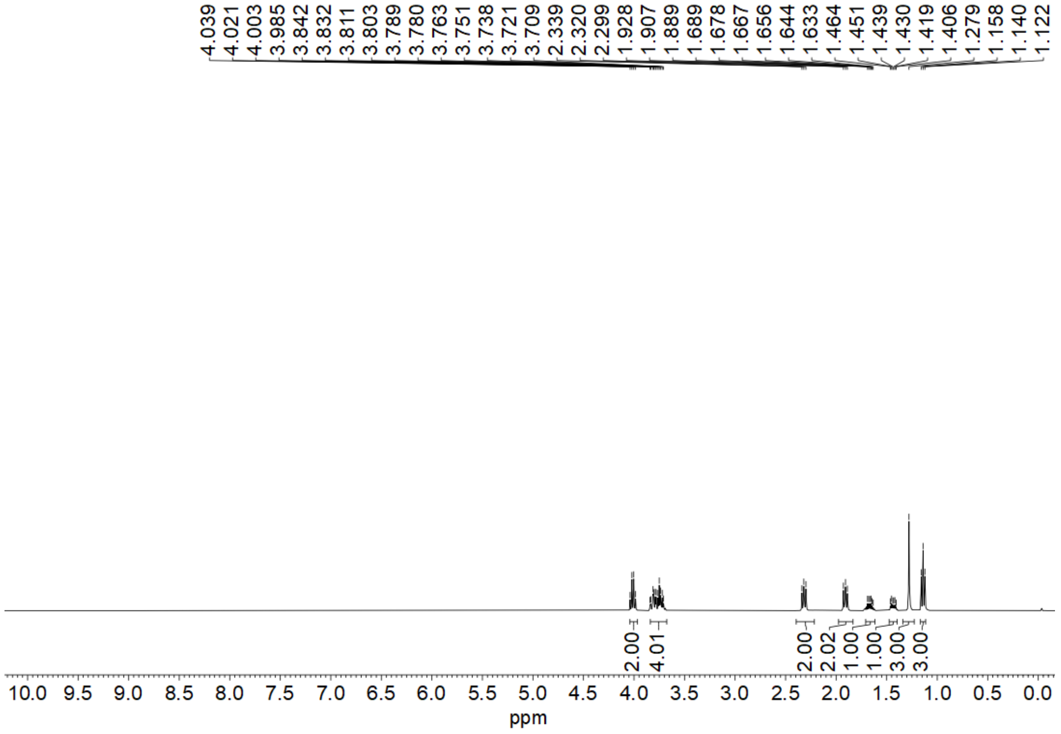

Figure S8. The ^{1}H-NMR spectrum of **LEK 2.**

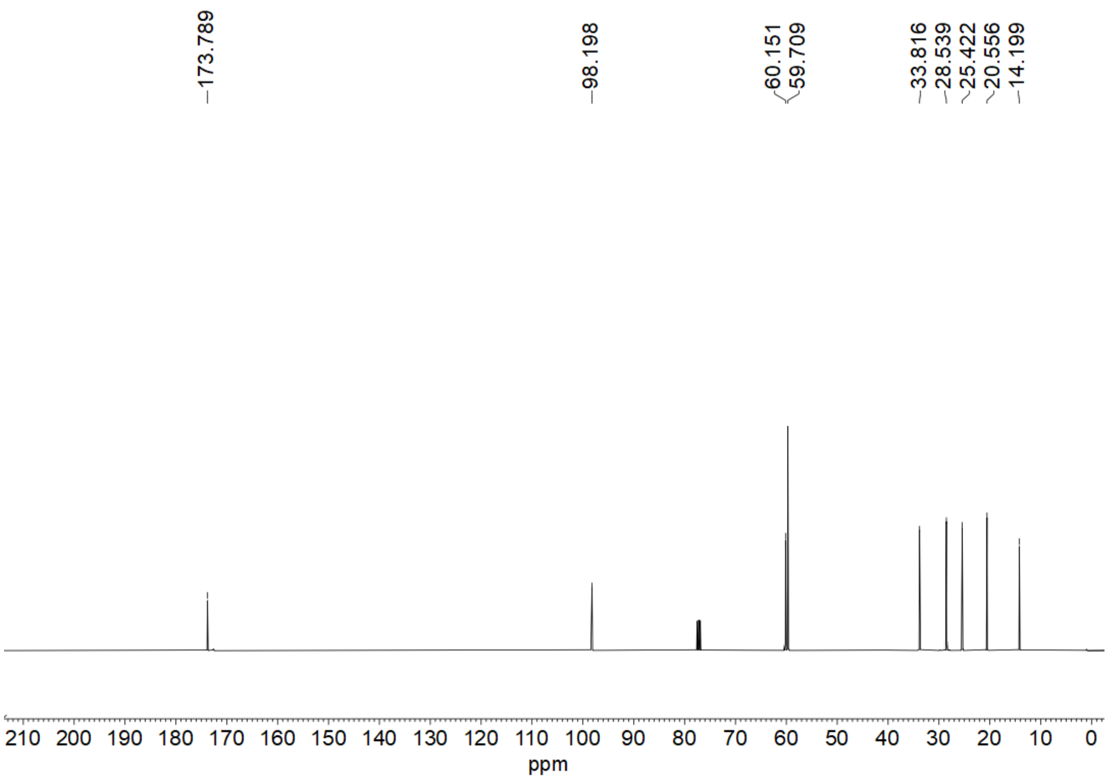

Figure S9. The ^{13}C-NMR spectrum of **LEK 2.**

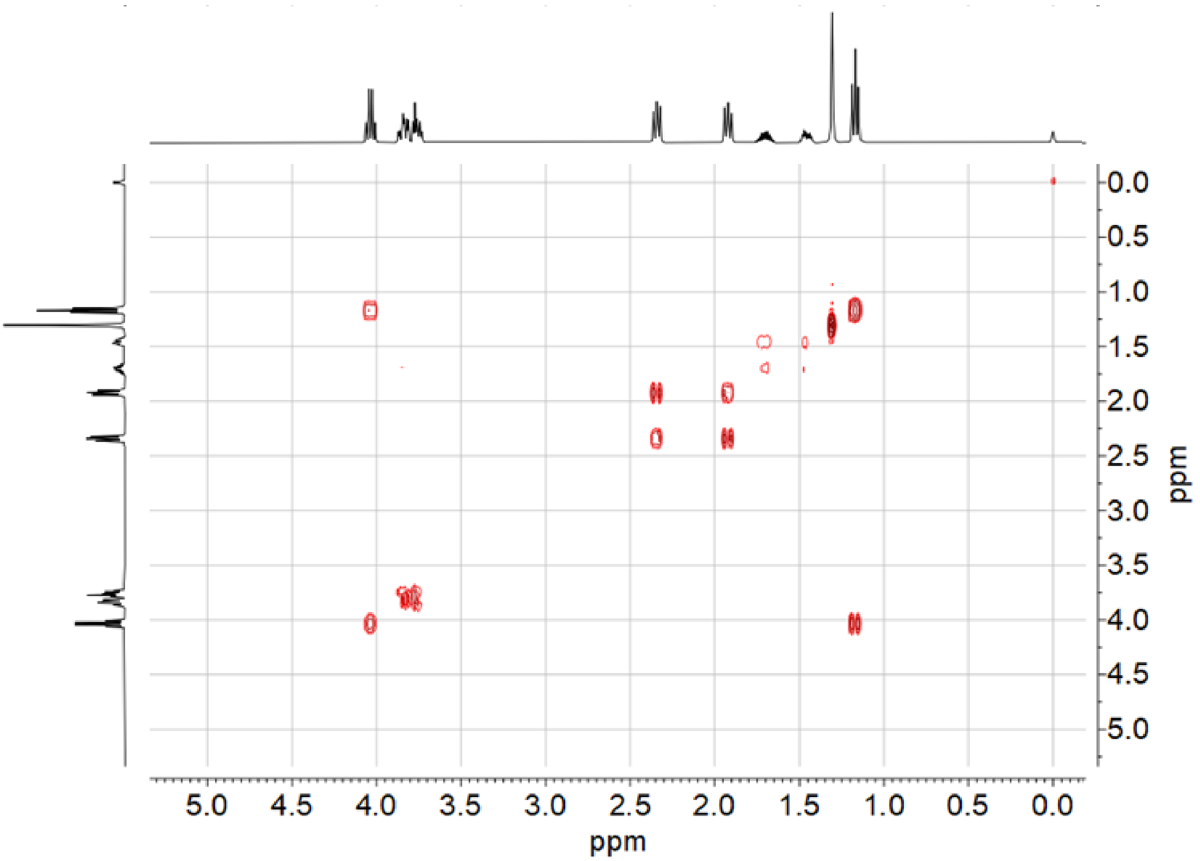

Figure S10. The ^{1}H-^{1}H COSY spectrum of **LEK 2.**

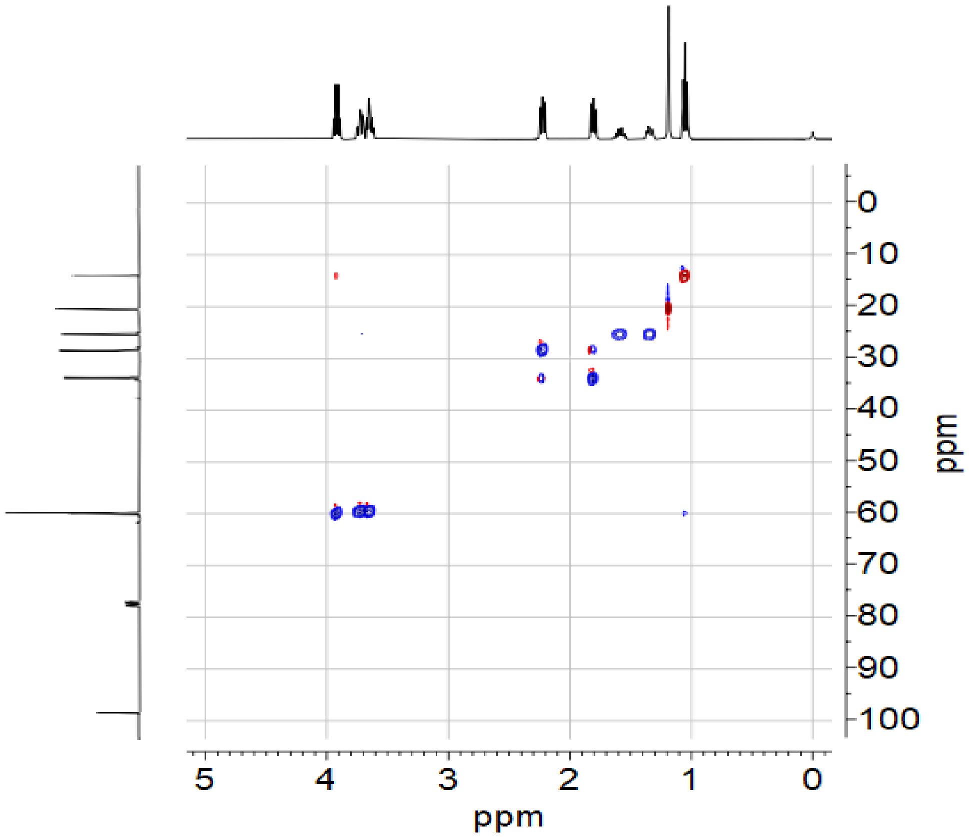

Figure S11. The HMQC spectrum of **LEK 2.**

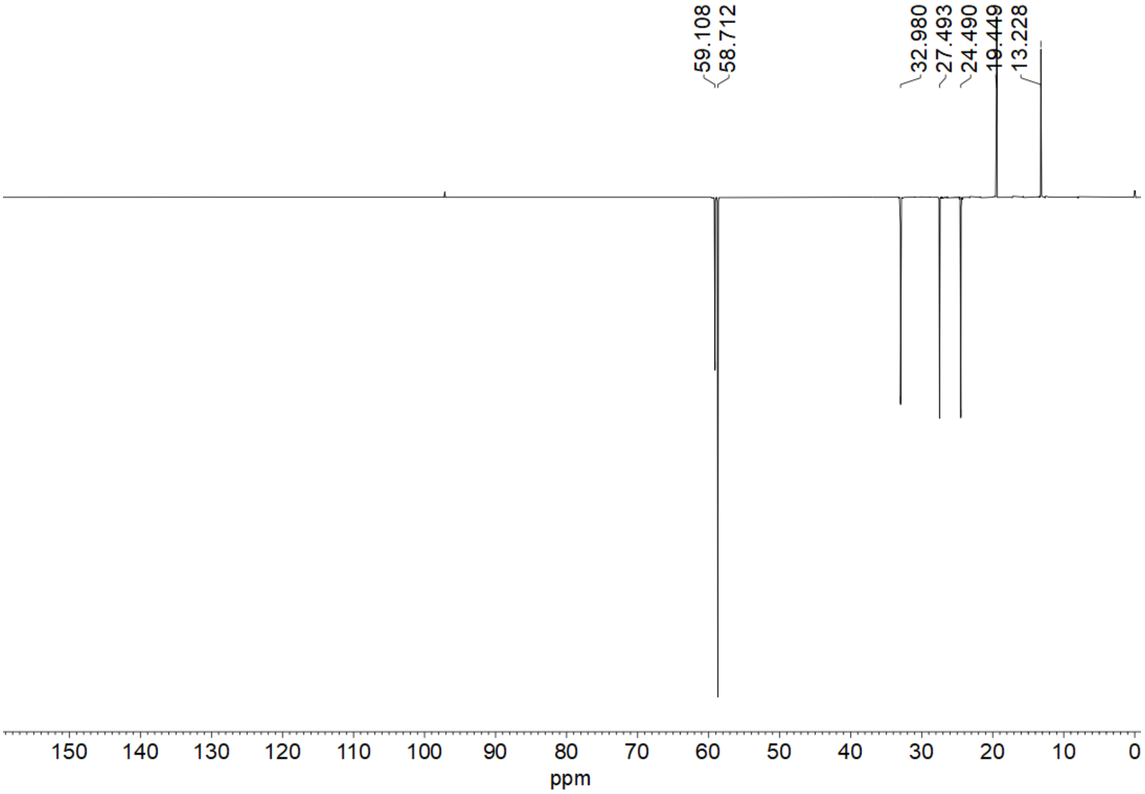

Figure S12. The DEPT-135 spectrum of **LEK 2.**

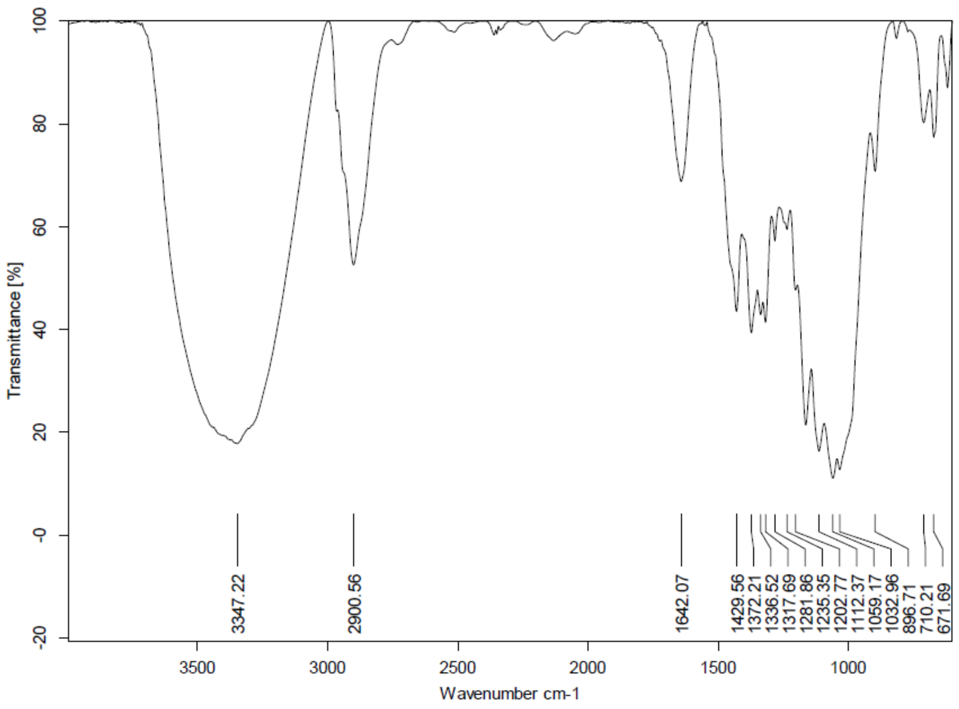

Figure S13. The FTIR spectrum of **CSA**.

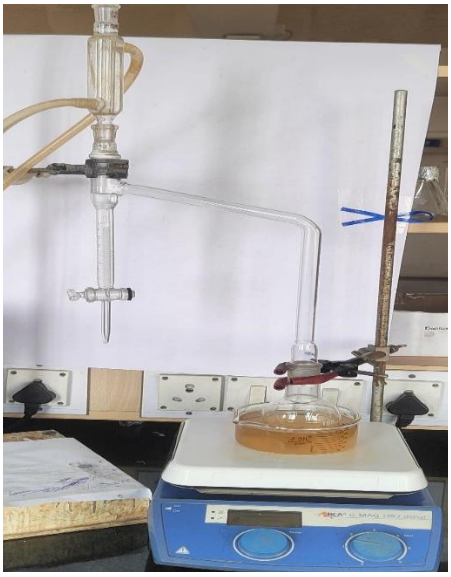

Figure S14. The experimental setup for synthesizing LEK (**1**&**2**) before wrapping with aluminum foil for insulation.

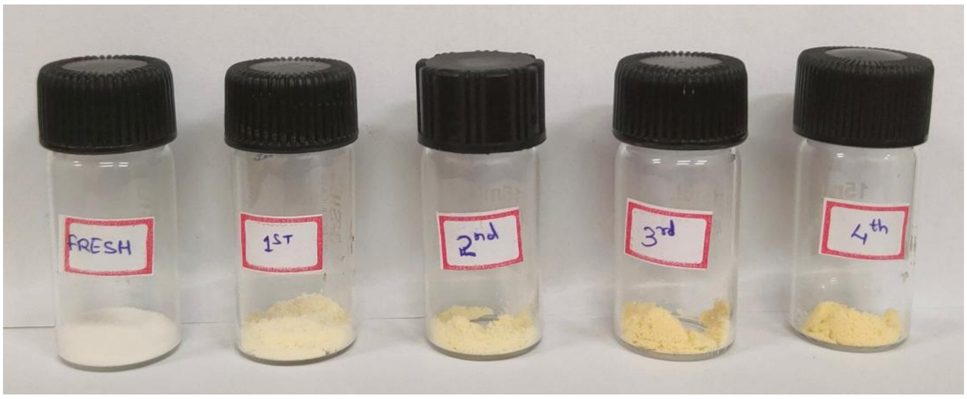

Figure S15. Photographic images of the fresh and recycled CSA catalyst samples.

References:

(1) Gundekari, S.; Mani, M.; Mitra, J.; Srinivasan, K. Selective Preparation of Renewable Ketals from Biomass-Based Carbonyl Compounds with Polyols Using β-Zeolite Catalyst. *Mol. Catal.* **2022**, *524*, 112269. https://doi.org/10.1016/j.mcat.2022.112269.
